# Copper Ions Absorbed on Acrylic-Acid-Grafted Polystyrene Enable Direct Bonding with Tunable Bonding Strength and Debonding on Demand

**DOI:** 10.3390/polym14235142

**Published:** 2022-11-25

**Authors:** Roman Günther, Walter Caseri, Christof Brändli

**Affiliations:** 1 Laboratory of Adhesives and Polymer Materials, Institute of Materials and Process Engineering, ZHAW Zurich University of Applied Sciences, 8401 Winterthur, Switzerland; 2 Multifunctional Materials, Department of Materials, ETH Zürich, 8093 Zürich, Switzerland

**Keywords:** debonding on demand, direct bonding, surface modification

## Abstract

Recycling adhesively bonded polymers is inconvenient due to its expensive separation and removal of adhesive residues. To tackle this problem, adhesive technologies are needed allowing debonding on demand and which do not contaminate the surface of the substrate. Direct bonding enabled by oxygen plasma treatment has already achieved substantial adhesion between flat substrates. However, debonding takes place by water, thus limiting the applications of this technology to water-free environments. The work presented in the following shows that this drawback can be overcome by grafting acrylic acid and adding copper(II) ions on the surface of polystyrene. In this process, the number of functional groups on the surface was significantly increased without increasing the surface roughness. The bonding strength between the substrates could be increased, and the process temperature could be lowered. Nevertheless, the samples could be debonded by exposure to EDTA solution under ultrasound. Hence, by combining acrylic acid grafting, variations in the bonding temperatures and the use of copper(II) ions, the bonding strength (5 N to >85 N) and the debonding time under the action of water can be tuned over large ranges (seconds to complete resistance).

## 1. Introduction

Bonding by adhesives is a widely used way of joining polymers. However, as a major drawback, the adhesives commonly cannot readily be debonded. This renders recycling virtually impossible, and as a consequence, the polymers have to be disposed of at the end of their life [1].

In order to overcome this problem, adhesive technologies have to be employed that allow debonding on demand. For example, hot melt adhesives were used for this purpose. At elevated temperatures, the adhesives soften, thus allowing them to debond easily [2]. As another possibility, functional fillers can be introduced into the adhesive matrix that expands when heated, weakening the adhesive bonds [3,4,5,6,7,8]. Further, adhesive bonds can be separated under the action of electricity [9] or light [10,11]. However, with the methods described above, adhesive residues remain at least on one side, which is not favorable for recycling the substrates.

In the case of polymer substrates, this disadvantage can be dealt with, for instance, by generating functional groups on the polymer surfaces that allow reversible bonding of the substrates [12]. Such groups can basically be created by UV irradiation [13,14], corona discharge [15,16,17], or plasma treatment [14,18,19,20,21]. Importantly, the resulting groups form a layer in the nanometer range; hence, their mass is negligible compared to the mass of the bulk material. Thus, they do not interfere in the recycling process.

In previous work, the effectiveness of this principle has been demonstrated on the example of smooth polystyrene (PS) surfaces treated with oxygen plasma to generate functional groups that allowed the substrates to bond directly, probably with hydrogen bonds playing a significant role in the adhesion [12]. The resulting adhesion was strong and even exceeded the strength of the substrates, leading to material failure instead of bond failure. Nevertheless, within seconds, the firm bond could be released entirely by treatment with water.

Unfortunately, debonding on demand with water severely limits the field of application of this bonding technology since related joints are only applicable in the absence of water. To counteract this disadvantage and broaden the field of application of this technology, we show further development of this technology in this work. In an additional process step, acrylic acid (AA) is grafted onto the surface to increase the density of functional groups on the surface. Further, the increased number of functional groups opens the possibility of complexing metal ions on the surface. Ligand–metal ion interactions can be more stable than hydrogen bonds. In fact, this effect was already applied to hydrogels [22,23,24,25,26]. In the following, the influence of grafting of acrylic acid on the topography and the impact of the bonding parameters on the bond strength, debonding time, and the stability of the functional groups on the surface were investigated.

## 2. Materials and Methods

Acrylic acid (Sigma Aldrich, St. Louis, MO, USA) was purified through a basic Al_2_O_3_ column. Copper(II) acetate (Cu(ac)_2_) monohydrate and CuCl_2_ dihydrate, from Sigma Aldrich, were heated to 120 °C for 10 min to remove crystal water. Absolute ethanol (Alcosuisse), ammonia solution 2.0 M in ethanol (Sigma Aldrich), ethylenediaminetetraacetic acid disodium salt (Disodium EDTA, Sigma Aldrich), and polystyrene (GP 585 X, from Synthos Chemical Innovations, Poland, M_n_ = 56,079 g/mol, M_w_ = 218,167 g/mol) were used as received.

### 2.1. Fabrication of Polymer Substrates

In the first step, preforms with dimensions of 80 × 10 × 4 mm^3^ were prepared from the polymer granules in an injection molding process using a BOY XS injection molding machine from Dr. Boy GmbH & Co., KG, Neustadt, Germany. The polymer was injected at 240 °C at 80 bar pressure. The specimens were then smoothed in a hot press (Atlas 15T with heated plates, Specac Ltd. Limited, Orpington, UK) between two silicon wafers (Dummy CZ-Si Wafer, MicroChemicals GmbH, Ulm, Germany) for 30 s at 180 °C and a load of 200 kg. The hot samples were cooled in the upright position at room temperature. The resulting 1.2 mm thick samples were cut to approximately 7 × 7 mm^2^ and 20 × 20 mm^2^ squares with a wire cutter.

### 2.2. Oxygen Plasma Treatment

Samples were treated in a Diener nano plasma furnace (Diener electronic GmbH + Co., KG, Ebhausen, Germany) in an oxygen atmosphere for 12 s at 0.2 mbar pressure and 200 W power. The plasma furnace was run empty for 5 min before each treatment for cleaning and minimizing contamination.

### 2.3. Acrylic Acid Grafting

The acrylic acid grafting was performed based on the procedure described by Alves et al. [27]. Plasma-treated polystyrene samples were placed in 3D-printed sample holders and placed in a Hellendahl staining box. For each staining box, 30 mL of 30 wt% aqueous acrylic acid solution was combined with 0.15 mL of 0.015 M FeSO_4_/0.005 M H_2_SO_4_ solution as catalyst. The chamber was closed airtight with a 3D-printed lid and clamp. The loaded staining chambers were placed in a 70 °C water bath. The reaction took place under a continuous N_2_ stream for 120 min. The acrylic-acid-grafted polystyrene samples were rinsed with distilled water to remove possible unreacted monomers and non-binding polymer chains.

### 2.4. Copper(II) Loading

A total of 0.1 M copper(II) solution was prepared by dissolving CuCl_2_ or Cu(ac)_2_ (ac = acetate) in water, ethanol, and anhydrous ammonia (2 M) in ethanol solution. The salts could be completely dissolved in all solvents. The surfaces were loaded with copper(II) ions by dropping the solutions onto the surfaces. After an exposure time of 10 s, the surfaces were rinsed with a washing bottle with 50 mL ethanol to remove excess ions. The samples were blown dry and stored in a dry atmosphere until further use. The washing procedure was optimized until a significant shift of copper in the Wagner plots was achieved. Therefore, most copper(II) was not present in crystalline form, and a high copper(II) loading was granted.

### 2.5. Atomic Force Microscopy (AFM)

AFM was performed with an NTEGRA AFM (NT-MDT Spectrum Instruments, Moscow, Russia) in semi-contact mode (tapping mode) and Nova Px 3.5.0 software. NGS01 tips from NT-MDT Spectrum Instruments with a typical tip radius of 6 nm were used. The scan parameters were optimized using the ScanT software extension in the attractive measurement regime. Scans were performed over an area of 10 × 10 µm^2^ and a resolution of 512 pixels × 512 pixels for each sample. Each measurement line was recorded in two measuring directions. Based on the two images, a minimum was calculated, minimizing the parachuting effect. Subsequently, the images were aligned using a first-order line fit. Surface roughness was calculated using the integrated roughness analysis over the whole surface of the 10 × 10 µm^2^ scans. The peaks were cut in some images to allow a better comparison of structures.

### 2.6. X-ray Photoelectron Spectroscopy (XPS)

XPS was performed with a SPECS^TM^ spectrometer (SPECS GmbH, Berlin, Germany) using a Mg Kα X-ray source (*λ* = 1253.6 eV) with a power of 300 W. The measurements were made at room temperature. Each sample was studied at one spot. The investigated area typically amounted to 7 × 10 mm^2^. Survey spectra were acquired over a binding energy range of 0–1000 eV at a pass energy of 30 eV and resolution of 0.5 eV/step. High-resolution spectra of C 1s were obtained as an average of three scans acquired at a pass energy of 20 eV and a resolution of 0.05 eV/step. The CasaXPS software was used for background subtraction (U 2 Tougaard-type), peak integration, quantitative chemical analysis, and deconvolution. The adventitious C 1s peak at 285 eV was used to calibrate the binding energy scale. The C 1s signals of the scans were fitted using a database [28] as a reference.

For the Wagner plots, high-resolution spectra of Cu 2p3/2 and Cu LMM signals were obtained as an average of three scans acquired with a pass energy of 20 eV. The bonding and kinetic energy maxima were evaluated for Cu 2p3/2 and Cu LMM, respectively. With the gathered maxima, the Wagener plots were created.

### 2.7. Adhesion Tests

Butt tensile tests were carried out with a centrifugal adhesion test analyzer (LUMifrac) from LUM GmbH (Berlin, Germany) at room temperature. The samples with grafted acrylic acid or untreated polymer samples were bonded with a two-component epoxy adhesive (Betamate 2090, DuPont) to a sample holder of 10 mm diameter. The aluminum sample holder was sandblasted and cleaned with acetone to ensure high bond strength with the adhesive. The thickness of the adhesive was adjusted to 0.2 mm with the aid of glass spheres. The adhesive cured for at least 24 h at room temperature, according to the manufacturer’s advice. Before joining, the acrylic-acid-grafted surfaces were rinsed with ethanol and blown dry, and the untreated samples were treated in oxygen plasma. The polymer substrates were then pressed together in a hot press at temperatures from 40 °C to 80 °C for 1 min at a load of 200 kg. The samples were kept at room temperature in a dry atmosphere until they were tested in the LUMifrac device. For testing, up to eight samples were loaded simultaneously in the measuring chamber. The increase in force was set to 1 N s^−1^. Through rotation, the applied centrifugal force yielded a nearly pure butt tensile load to the specimen. At bonding failure, the copper weight triggered the sensor, and the centrifuge rotation speed with the corresponding force at failure was recorded.

### 2.8. Debonding Experiments

To observe debonding, samples were examined in a Petri dish under a digital microscope (VHX-6000 V3.0.0.116, Keyence, Osaka, Japan). The sample was pressed down with tweezers to prevent floating away. After starting the recording, the Petri dish was filled with water. The debonding was filmed at 15 frames/s for the first 10 min. Subsequently, frames of the samples were recorded at 2 min intervals. Based on the recordings, the debonding time was evaluated.

## 3. Results and Discussion

### 3.1. Modification of the Polystyrene Surfaces

**Figure 1:**
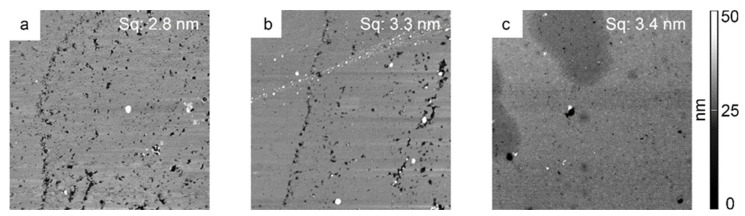
AFM images of polystyrene before and after oxygen plasma treatment and after acrylic acid grafting. The edge length of all AFM images is 10 µm. (**a**) Polystyrene surface after hot pressing between two silicon wafers, before plasma activation, surface roughness Sq = 2.8 nm. (**b**) Polystyrene surface after plasma activation, Sq = 3.3 nm. (**c**) Polystyrene surface after plasma treatment and additional grafting with acrylic acid, Sq = 3.4 nm. The peaks were cut in the images to allow a better comparison of structures.

Since only short-range intramolecular forces act between the substrates when joining the surfaces directly together, the surfaces must be as smooth as possible. Therefore, very flat and smooth polystyrene surfaces were prepared by injection molding with consecutive hot pressing between two silicon wafers. Functional groups were introduced to the surface by oxygen plasma treatment and by the grafting of acrylic acid, according to the work of Alves et al. [27]. The surface roughness before and after the individual treatment steps was investigated by AFM (Figure 1). The roughness of the polystyrene surfaces (about 3 nm) did not change considerably upon plasma treatment and subsequent grafting with acrylic acid. It was found [12] that this surface roughness is suited for the direct bonding of substrates.

**Figure 2:**
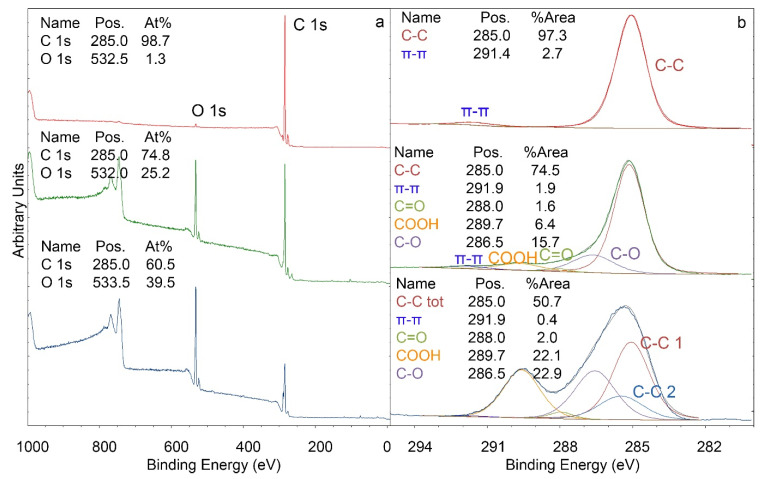
XPS data before (red curve) and after oxygen plasma treatment (green curve), and after acrylic acid grafting (blue curve). (**a**) Survey spectra, (**b**) high-resolution scan of the C 1s signal. C–C tot corresponds to the sum of C–C 1 and C–C 2. The spectra were normalized (min to max).

XPS studies (Figure 2) show that the oxygen concentration on the surface increased strikingly upon exposure to oxygen plasma treatment from 1 At% to 25 At%. A more detailed investigation of the C 1s signal provides information on the nature of the additional oxygen atoms. Additional C–O (16% of C 1s), C=O (2% of C 1s), and COOH (6% of C 1s) groups were detected. By subsequent grafting of acrylic acid, the oxygen concentration increased to 40 At%. A detailed look at the C 1s signal reveals an increase in COOH groups (22% of C 1s vs. 6% of C 1s). Furthermore, the proportion of C–O groups increased to 23% of C 1s. The amount of C=O groups remained essentially constant. The increase in COOH groups shows that grafting with acrylic acid was successful, and substrates with the indicated quantities of functional groups were used for the experiments below (as a side remark, batches with different amounts of functional groups than indicated above were sometimes also obtained with the method of Alves et al. [27]; these batches, however, were not used for further studies).

### 3.2. Loading of Modified Polystyrene Surfaces with Copper(II) Ions

**Figure 3:**
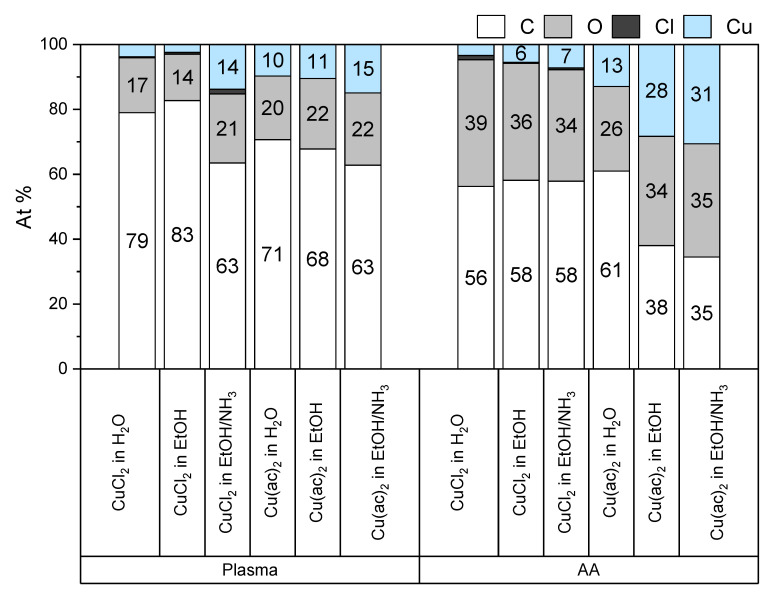
XPS data of the atomic composition (in At%) of surface-modified polystyrene samples after exposure to dissolved copper(II) salts. Plasma = plasma-treated, AA = acrylic-acid-grafted surface.

The acrylic-acid-modified substrates were exposed to copper(II) salts with the aim of binding as many copper(II) ions to the respective surfaces as possible. Copper(II) was chosen because it forms the most stable complexes of the bivalent ions of the first transition metal period according to the Irving–Williams series [29,30]. Substrates were treated with aqueous or ethanolic solutions of Cu(Cl)_2_ and Cu(ac)_2_. Water and ethanol showed sufficient solubility to completely dissolve CuCl_2_ and Cu(ac)_2_. After solution treatment, the samples were washed with 50 mL ethanol to remove excess copper(II) ions that did not interact considerably with the surface of the substrates. The surfaces were examined with XPS, and the results are shown in Figure 3.

To achieve an ideal washing, where the copper(II) ions are essentially not present in adsorbed salt while maintaining high copper(II) loadings, the washing procedure was optimized using Wagner plots. Wagner plots visualize values of kinetic energies of a specific Auger peak against the measured binding energy. When a significant shift occurs, a change in the compound can be presumed [31].

A low copper(II) loading with CuCl_2_ in water and ethanol was obtained for the plasma-treated (4 At% and 3 At%, respectively) and the acrylic-acid-grafted (3 At% and 6 At%, respectively) substrates. With Cu(ac)_2_ in water and ethanol, the copper(II) loading for plasma-treated substrates amounted to 10 At% and 11 At%, respectively, and for acrylic-acid-grafted substrates to 13 At% and 28 At%, respectively. Thus, the highest loadings were obtained with Cu(ac)_2_ applied in ethanol on surfaces modified with acrylic acid.

It is possible to deprotonate carboxylic acid groups to increase their affinity towards metal ions [30]. However, a high pH value should be avoided for this purpose in an aqueous solution to prevent the precipitation of Cu(OH)_2_ [32]. Accordingly, ammonia was used in anhydrous ethanol solutions of the copper(II) salts. Although the ammonia forms complexes with copper(II) ions, the loading of copper(II) ions on plasma-treated samples increased markedly to 14 At% in the case of CuCl_2_ and slightly to 15 At% in the case of Cu(ac)_2_. However, when acrylic-acid-grafted surfaces were employed, the copper(II) loading did not increase considerably compared to the pure ethanolic solutions (7 At% and 31% for CuCl_2_ and Cu(ac)_2_, respectively). For the experiments described below, samples were loaded with Cu(ac)_2_ in ammonia/ethanol solutions, which provide a high loading of copper(II).

**Figure 4:**
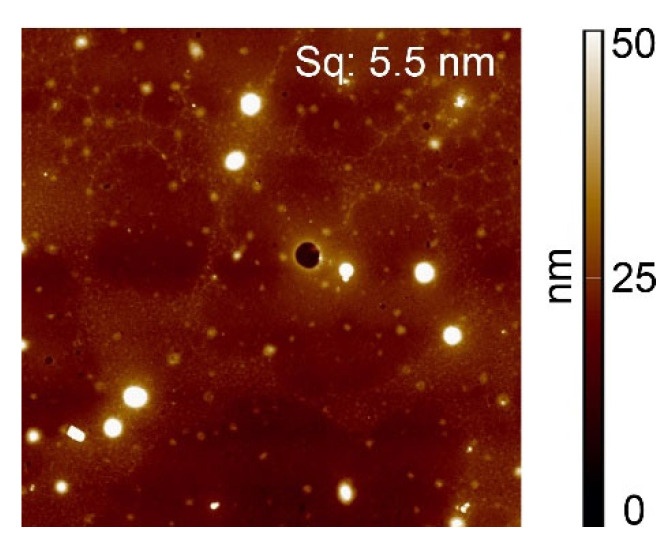
AFM images of polystyrene after loading with copper(II). The edge length of all AFM images is 10 µm. Polystyrene surface exposed to 0.1 M Cu(ac)_2_ in ethanol/NH_3_ solution, surface roughness Sq = 5.5 nm.

**Figure 5:**
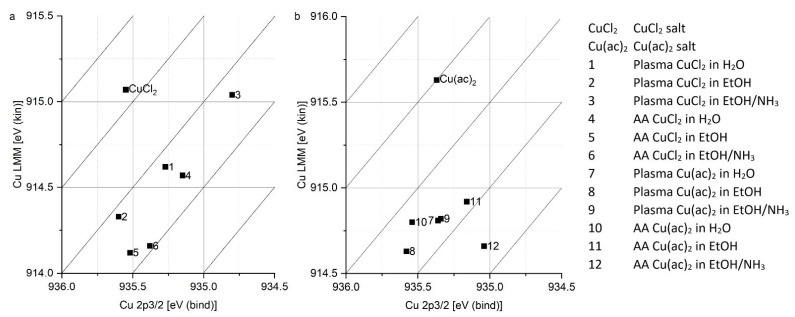
Wagner plots of the Cu 2p3/2 signal and the Cu LMM Auger signal, retrieved from XPS data. (**a**) CuCl_2_ and surfaces loaded with CuCl_2_. (**b**) Cu(ac)_2_ and surfaces loaded with Cu(ac)_2_.

The topography of the acrylic-acid-grafted surfaces loaded with Cu(ac)_2_ in ammonia solution in ethanol (31% Cu) was examined with AFM (Figure 4). The surface roughness (Sq = 5.5 nm) was somewhat higher compared to the value in the absence of copper (Sq = 3.4 nm), essentially due to the formation of round objects with diameters ranging from 10 nm to 400 nm. It might be that the surface of the grafted polystyrene has rearranged to some extent due to ion-induced nano structuration [33] or that small Cu(ac)_2_ particles formed. Yet in the samples exposed to CuCl_2_ solutions, only a small amount of Cl atoms needed for the salt formation was found on the surfaces, which is not in agreement with substantial formation. Moreover, Wagner plots of copper(II)-loaded surfaces (Figure 5) reveal distinct shifts in energies for all samples with respect to CuCl_2_ and Cu(ac)_2_, which implies that CuCl_2_ and Cu(ac)_2,_ are not present to a considerable extent on the surfaces exposed to the respective salts, in particular for those exposed to Cu(ac)_2_ which were used for further experiments.

### 3.3. Adhesion Experiments

**Figure 6:**
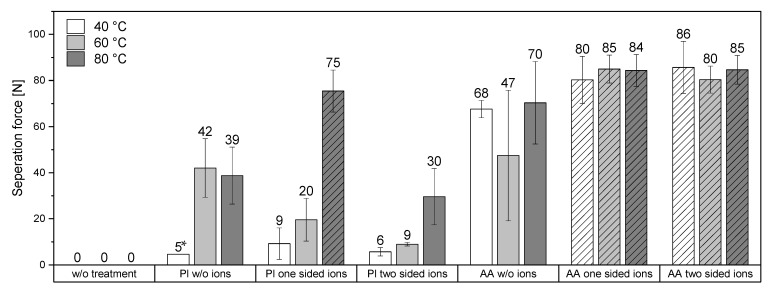
Separation force of bonded substrates, measured by a LUMifrac device, at different temperatures (40 °C, 60 °C, and 80 °C) and with various surface treatments. Pl = plasma-treated, AA = acrylic-acid-grafted surface, *w*/*o* ions = without ions, one sided ions = only one of the two bonded substrates had been loaded with Cu ions, two sided ions = both sides of the bonded substrates had been loaded with Cu ions. Hatched boxes = cohesive failure, i.e., the real separation force is higher but not measurable. Unless otherwise indicated, five samples per experiment were tested. * = only one out of seven samples bonded considerably.

Bonding experiments were performed by pressing the substrates together at temperatures between 40 °C and 80 °C. The adhesion measurements were carried out using a LUMifrac centrifugal adhesion test analyzer (for details see Section 2.2). For testing, up to eight samples were loaded simultaneously in the measurement chamber. Through rotation, the applied centrifugal force yielded a nearly pure butt tensile load to the specimen. At bonding failure, a copper weight triggered a sensor, and the centrifuge rotation speed with the corresponding force at failure was recorded. Figure 6 shows the results of the LUMifrac adhesion measurements. Without surface pretreatment of the samples, the adhesive forces between the substrates were below the detection limit at 40 °C, 60 °C, and 80 °C. The adhesion of the substrates exposed to oxygen plasma was higher when they were bonded together at 60 °C or 80 °C, in line with previous observations [12]. This is probably caused by the plastic deformation of the polymer surfaces at higher temperatures and under the pressure applied during the bonding. This minimal plastic deformation could be sufficient to compensate minor irregularities and thus increases the contact area accessible to adhesive forces.

Copper(II) ions on plasma-treated surfaces did not significantly increase the adhesion, except for the unilaterally loaded specimens at 80 °C bonding temperature. Here, the adhesion forces achieved were so strong that the samples failed within the polystyrene substrates rather than in the bonding zone. Therefore, the real adhesive force was between the substrates could not be determined more specifically.

Substrates with surfaces grafted with acrylic acid consistently revealed high adhesion, regardless of the bonding temperature. Notably, those surfaces possess more functional groups than plasma-treated surfaces, which promotes the bonding of the substrates. Further, the grafting of acrylic acid created flexible groups on the surface. The enhanced mobility allows the functional groups to arrange themselves and bridge unevenness, which could increase the direct contact area and thus improve adhesion.

Interestingly, copper(II) ions enhanced the adhesion of the samples with acrylic-acid-grafted surfaces at all temperatures and when one or both substrates had been loaded with copper(II). In all those cases, cohesive failure was observed (with adhesion forces above 80 N–86 N) at least 90 N, i.e., the samples broke within one of the substrates and not at the interface between the substrates, in contrast to the corresponding specimen without copper(II). Compared to the plasma-treated samples, the acrylic-acid-grafted samples possess more functional groups, particularly carboxylate groups, which coordinate well with copper(II), thus increasing the adhesion of carboxylate groups of both surfaces coordinated to the same copper ion.

### 3.4. Debonding

In order to investigate whether and how fast the substrates can be debonded from each other, samples were placed in Petri dishes under a microscope equipped with a film camera at room temperature. After starting the recording, the Petri dish was filled with water. The first 10 min of the debonding process were filmed with 15 frames/s. Subsequently, frames were taken in 2 min intervals. Based on the recordings, the debonding time was evaluated (Table 1).

The plasma-treated samples bonded at 40 °C without copper(II) ions debonded after 10 s. When bonded at 80 °C, the debonding time increased to 121 s. The loading of copper(II) ions to one surface increased the debonding time to 1130 s for bonding at 40 °C. The samples bonded at 80 °C did not separate during the maximum observation time of 18 h (65,000 s) in water. By loading both surfaces with copper(II) ions, the samples separated after 722 s and 1062 s for bonding at 40 °C and 80 °C, respectively. Acrylic-acid-grafted samples without copper(II) ions debonded after 64 s when bonded at 40 °C and after 1320 s when bonded at 80 °C. The unilateral loading with copper(II) ions increased the debonding time to 820 s when bonded at 40 °C. The debonding did not occur within 18 h (65,000 s) when the surfaces were bonded at 80 °C. When both acrylic-acid-grafted surfaces were loaded with copper(II) ions and bonded at 40 °C and 80 °C, the debonding time decreased to 45 s and 37 s, respectively.

Thus, at first glance, the debonding time increases strongly with increased bonding temperature for almost all cases. Only the acrylic-acid-grafted samples with bilateral ion loading decreased in debonding time. The prolonged debonding time for specimen bonded at 80 °C, can be explained by the better bonding of the surfaces due to plastic deformation and the increased mobility of the functional groups. For unilateral loading with copper(II), the bonding time increased and debonding did not occur within the timeframe of the experiment for the plasma-treated and acrylic-acid-treated substrates. As a possible explanation, treatment of both surfaces with copper(II) ions might lead to a coordination sphere of a given copper(II) ion which is constituted by ligands on one surface alone instead of a ligand sphere of units that are part of both surfaces. Accordingly, the debonding time decreased when bilateral loading of copper(II) was applied compared to unilateral loading for all tested samples.

In order to investigate if the strongly enhanced debonding time can indeed be associated with the presence of copper(II) ions, the plasma-treated and acrylic-acid-treated samples, which could not be debonded after 65,000 s (cf. Table 1) were exposed to a 1 g/L ethylenediaminetetraacetic acid disodium salt (disodium EDTA) solution under ultrasonication. EDTA forms very strong complexes with copper(II) ions, and it is expected that those ions are removed from the surface accordingly. Indeed, under these circumstances, debonding took place within 1800 s. Thus, importantly, although the respective bonds show good resistance towards water, they can be debonded on demand simply by using EDTA.

### 3.5. Durability of the Surface Functionalization

To determine the durability of the surface functionalization, surfaces were examined with XPS after extensive washing with deionized water and after debonding in water. The resulting atomic composition of the surfaces and the functional groups are shown in Table 2. After washing the plasma-treated samples, the oxygen contents and the functional groups decreased moderately. Thus, it seems that some of them were washed away. This could be explained by the fact that plasma treatment frequently produces low molecular weight oxidized material (LMWOM) which can be washed away [34]. Comparing the surfaces after washing and debonding, similar quantities of functionalities are lost in both processes, i.e., it can be concluded that LMWOMs were removed by water also after debonding.

The acrylic-acid-grafted samples were already washed in the grafting process, yet after debonding, the oxygen concentration on the surface, especially the number of COOH groups, decreased strongly. Maybe the mechanical stress during bonding and debonding induced the breaking of bonds, leading to low molecular weight fragments that can be washed away.

In summary, it is evident that more functional groups are available in all cases when acrylic acid is additionally grafted onto the surface. The overall higher number of functionalities gives the acrylic-acid-grafted surfaces a clear advantage over those treated only with oxygen plasma.

## 4. Conclusions

By grafting with acrylic acid, the number of functional groups could be increased on polystyrene surfaces treated with oxygen plasma, which allowed us to study the influence of this surface modification on adhesive properties. Better adhesion was achieved with acrylic-acid-modified surfaces at lower bonding temperatures. In addition, acrylic acid grafting allowed loading with larger amounts of copper(II) ions. These copper(II) ions could be loaded in higher concentrations on the acrylic-acid-modified surfaces than with plasma-treated surfaces alone, leading to increased adhesion.

Notably, the presence of copper(II) ions allowed tuning the debonding time in water over several orders of magnitudes. By choosing unilateral or bilateral loading of the bonded substrates and the bonding temperature, the debonding time varied from seconds to at least 18 h, where no debonding was achieved within the observation time. However, these samples could be separated readily by exposure to an EDTA solution, which is attributed to the formation of copper(II)–EDTA complexes and, thus, to the annihilation of coordination bonds between copper(II) ions and functional groups of the modified polystyrene surfaces.

## Figures and Tables

**Table 1:** Debonding time of various samples upon immersion in water at room temperature, w/o ions = without ions, one sided = only one of the two bonded substrates had been loaded with copper(II) ions, two sided = both sides of the bonded substrates had been loaded with copper(II) ions. The temperature represents the bonding temperature of the samples. One sample per experiment was tested.

		w/o Ions [s]	One Sided [s]	Two Sided [s]
plasma-treated	40 °C	10	1130	722
	80 °C	121	>65,000	1062
acrylic-acid-treated	40 °C	64	820	45
	80 °C	1320	>65,000	37

**Table 2:** XPS measurement. Atomic composition and functional groups of surface-treated polystyrene before and after washing and after debonding.

	C [At%]	O [At%]	C–C [%]	C–O [%]	C=O [%]	O–C=O [%]	π–π [%]
PS Plasma	75	25	75	16	2	6	2
PS Plasma washed	82	18	77	16	1	2	3
PS Plasma after debonding	81	19	76	14	2	5	3
PS AA washed	61	40	51	23	2	22	0
PS AA after debonding	74	26	63	22	3	10	1

## Data Availability

Not applicable.
